# Supplementary material for: Self-Reported Dyslexia Traits as Positive Predictors of Self-Reported Cognitive Failures in the Workplace
Source: Behav Sci (Basel). 2025 Nov 18;15(11):1582. doi: 10.3390/bs15111582 (PMC12649733; doi:10.3390/bs15111582)
Supplement: Supplementary file 1 [file behavsci-15-01582-s001.zip › behavsci-3880434-supplementary.pdf]

Table S1

The intercorrelations between the predictor variables.

Predictor variable	1.	2.	3.	4.	5.	6.	7.	8.	9.	10.	11.	12.	13.	14.	15.
1. Age	-														
2. MPED Busyness	-.093	-													
3. MPED Routine	.067	-.247 ***	-												
4. BFI-10 Extraversion	.028	.035	-.116 *	-											
5. BFI-10 Agreeableness	.044	-.095	.096	.144 **	-										
6. BFI-10 Conscientiousness	.195 ***	.011	.132 **	.106 *	.271 ***	-									
7. BFI-10 Neuroticism	-.039	.129 **	-.065	-.117 *	-.310 ***	-.173 ***	-								

8. BFI-10 Openness	-.050	.021	-.040	.180***	-.006	.016	-.025	-							
9. SWEMBS total score	.152**	-.218***	.187***	.277***	.326***	.461***	-.381***	.016	-						
10. ASRS score	-.199***	.392***	-.203***	-.105*	-.273***	-.412***	.212***	.139**	-.498***	-					
11. ARQ score	-.113*	.182***	-.134**	-.170***	-.155**	-.316***	.188***	-.005	-.433***	.631***	-				
12. Total WCFS Score	-.120*	.394***	-.134**	-.118*	-.282***	-.435***	.265***	.056	-.471***	.770***	.635***	-			
13. WCFS Memory	-.150**	.375***	-.120*	-.159***	-.249***	-.361***	.227***	.037	-.434***	.720***	.610***	.899***	-		
14. WCFS Attention	-.076	.322***	-.096	-.108*	-.288***	-.447***	.234***	.071	-.423***	.709***	.566***	.900***	.709***	-	
15. WCFS Action	-.092	.349***	-.144**	-.033	-.193***	-.324***	.241***	.033	-.383***	.587***	.491***	.836***	.660***	.612***	-

Key: * $p \leq .05$, ** $p < .01$, *** $p < .001$
